# Nested PCR for the Diagnosis of Feline Sporotrichosis From Formalin-Fixed and Paraffin-Embedded Samples Using Different DNA Extraction Protocols

**DOI:** 10.3389/fvets.2021.755897

**Published:** 2022-01-05

**Authors:** Raul Leal Faria Luiz, Rodrigo Caldas Menezes, Sandro Antonio Pereira, Raquel de Vasconcellos Carvalhaes de Oliveira, Manoel Marques Evangelista Oliveira

**Affiliations:** ^1^Laboratory of Taxonomy, Biochemistry and Bioprospecting of Fungi, Oswaldo Cruz Institute, Oswaldo Cruz Foundation, Rio de Janeiro, Brazil; ^2^Laboratory of Clinical Research on Dermatozoonoses in Domestic Animals, Evandro Chagas National Institute of Infectious Diseases, Oswaldo Cruz Foundation, Rio de Janeiro, Brazil; ^3^Clinical Epidemiology Laboratory, Evandro Chagas National Institute of Infectious Diseases, Oswaldo Cruz Foundation, Rio de Janeiro, Brazil

**Keywords:** cats, *Sporothrix* sp., FFPE samples, DNA extraction, molecular diagnosis

## Abstract

Sporotrichosis is a chronic, cosmopolitan granulomatous mycosis that affects humans and animals. The infection is caused by the dimorphic fungi *Sporothrix* sp. The aims of the present study were to evaluate, standardize and validate a nested PCR technique using two DNA purification kits for the extraction of DNA from formalin fixed and paraffin-embedded tissues (FFPE) for *Sporothrix* sp. detection. FFPE mycological culture pellet samples of different *Sporothrix* species (*S. chilensis, S. mexicana, S. pallida, S. globosa, S. brasiliensis and S. schenckii*) were used as positive controls and clinical FFPE tissue samples of animals positive for *Cryptococcus* sp., *Leishmania infantum* and *Histoplasma* sp. were used as negative controls. Ten clinical FFPE skin samples from cats with sporotrichosis were used to validate the nested PCR. These samples were cut into two distinct paraffin sectioning protocols (5 and 16 μm thick). The paraffin sections were subjected to two different DNA extraction kits (chemical and thermal extractions). A nested PCR was performed on the extracted DNA to identify the genus *Sporothrix*. The chemical extraction protocol with the 5 μm thick paraffin section was more effective in extracting DNA from *Sporothrix* sp. from FFPE samples and the nested PCR technique showed the highest sensitivities (100% in the positive controls and of 50% in the skin samples of cats) and specificity (100%). Therefore, the nested PCR using this protocol has great potential to be applied in *Sporothrix* sp. diagnosis in FFPE samples of cats.

## Introduction

Sporotrichosis is a chronic cosmopolitan granulomatous mycosis, caused by the thermodimorphic fungus of the genus *Sporothrix*, which affects humans and a wide variety of animals, especially cats and dogs (1–4). Since 1998, there has been a large number of cases of sporotrichosis in Rio de Janeiro. Approximately 5,000 cases of sporotrichosis in humans (1998–2015), 5113 cases in cats (1998–2018), and 244 in dogs (1998–2014) were registered at the Evandro Chagas National Institute of Infectious Diseases (INI) of the Oswaldo Cruz Foundation (Fiocruz), one of the main reference center for the treatment of the disease in Brazil (3, 5, 6). The classical transmission of sporotrichosis occurs via traumatic implantation of vegetal or organic matter of soil contaminated with *Sporothrix* sp. conidia into the skin (7). In addition, zoonotic transmission through infected animals can occur (8). In most cats and dogs, the infection is acquired after fights with other infected cats and a contaminated environment (3, 6). Sporotrichosis is caused by pathogenic species of *Sporothrix*, including *S. brasiliensis, S. schenckii sensu stricto (s. str.), S. globosa, S. luriei, S. pallida, S. mexicana, and S. chilensis* (9–15). *S. schenckii* and *S. globosa* are the species generally associated with the classical (non-zoonotic) transmission route, while the species *S. brasiliensis* is associated with zoonotic sporotrichosis transmitted by cats (11, 15–18) in Brazil, mainly in Rio de Janeiro (19, 20), and in Argentina (21) and Paraguay (22). Cats are the animal species most affected by sporotrichosis caused by *S. brasiliensis*. This disease can lead to death of cats and its main clinical signs in these animals are multiple skin nodules and ulcers, mucosal lesions, enlarged lymph nodes and respiratory signs (20). The transmission of *S. brasiliensis* from an infected cat to humans, cats and dogs occurs through the bite, scratch, or contact with the exudate of cutaneous lesions (22).

The reference standard method for diagnosing animal sporotrichosis is mycological culture from clinical samples, such as biopsies or exudate from animal skin lesions or nasal swabs, with a presumptive diagnosis using microscopic examinations, such as cytopathology and histopathology (23). However, fungal growth in culture may not be observed and there may be microbiological contamination. It is a laborious and often time-consuming methodology, in addition to requiring considerable knowledge for the correct morphological identification of fungal species (24, 25). Formalin-fixed and paraffin-embedded (FFPE) tissue specimens represent an extremely valuable sample source for prospective and retrospective studies (26). If mycological culture is not possible for logistical or technical reasons, and if there are no alternative samples available other than FFPE material, polymerase chain reaction (PCR)-based molecular diagnostic approaches may be used (27).

PCR methodologies have recently been developed with the aim of improving the sensitivity and specificity of diagnostic tests for sporotrichosis. In addition, PCR allows the rapid identification of pathogenic *Sporothrix* sp., which is critical for the early treatment of sick cats, increasing the chances of clinical cure and reducing the risk of zoonotic transmission of *Sporothrix* sp. by these animals (28, 29). For the molecular identification of *Sporothrix* sp., PCR-based methodologies such as RFLP (restriction fragment length polymorphism) (30, 31), RAPD (random amplified polymorphic DNA) (32), PCR M13 fingerprinting and PCR T3B fingerprinting (33–35), partial DNA sequencing of the transcription region internal ribosomal RNA (ITS) (36), partial DNA sequencing of the calmodulin (CAL) (11, 37, 38), β-tubulin (βtub) (37) and chitin synthase (CHS) region (11, 39), nested PCR (40, 41) and PCR targeting the topoisomerase II gene (42, 43) have been developed. In these methodologies, DNA sequences in genomic loci encoding proteins, such as CAL (11, 37, 38), βtub (37) and CHS (11), in addition to another biomarker such as the ITS region (36) are used as molecular markers to differentiate between *Sporothrix* species. In cats, some of these PCR-based techniques were successfully applied to detect *Sporothrix* sp. in fungal isolates obtained by culture of clinical fresh samples (16, 20, 44) or directly from fresh clinical samples (45, 46). Direct detection of *Sporothrix* in FFPE skin samples from cats was only described by Bernhardt et al. (47), who used a fungal broad-range PCR assay targeting the internal transcribed spacer 2 (ITS 2) followed by amplicon sequencing. However, the sensitivity of this test was not evaluated (47).

Obtaining high-quality and pure PCR products from DNA extracted from FFPE tissue is a difficult task, because, in general, DNA in this material is scarce, degraded and contains substances that inhibit the amplification reaction, such as formalin, or inhibit proteinase K used in the extraction procedure, such as xylene (48). Prior to this study, the detection of *Sporothrix* sp. DNA by nested PCR in FFPE tissue samples was investigated only once, but using human samples (49, 50). There is still no standardized and reproducible nested PCR methodology for the definitive diagnosis of animal sporotrichosis using FFPE tissues. The aims of the present study were: to analyze and standardize a nested PCR technique for the detection of *Sporothrix* sp. in FFPE *Sporothrix* spp. culture pellets, using two kits for DNA extraction (chemical and thermal extractions) and two different types of paraffin sections; to validate the results using clinical FFPE samples from cats; and to identify at the genus-level all pathogenic species of *Sporothrix* and clinical samples by nested PCR.

## Methods

### Samples

Pellets from isolates of six different reference strains of *Sporothrix* species characterized in previous studies were used (*S. chilensis, S. mexicana, S. pallida, S. globosa, S. brasiliensis* and *S. schenckii*) (33, 51–53). The pellets were obtained by centrifugation of mycological culture and subsequent fixation in 10% buffered formalin and embedding in paraffin (54). These paraffin blocks (FFPE) were prepared in 2019. FFPE tissue samples from mandibular lymph nodes of a *Cryptococcus* sp. positive cat, and from skin and spleen of *Leishmania infantum* and *Histoplasma* sp. positive dogs, respectively, were used to evaluate specificity and the occurrence of cross-reactions. These FFPE tissues were from 2013, 2016 and 2013, respectively. To validate our results, clinical FFPE skin samples from ten cats with diagnosis of sporotrichosis by the isolation of *Sporothrix* sp. in mycological culture were used. Two of these FFPE skin samples were from 2009, four samples were from 2015 and four samples were from 2017. These cats were randomly selected from the cohort treated at the Laboratory of Clinical Research in Dermatozoonosis in Domestic Animals, Evandro Chagas National Institute of Infectious Diseases, Oswaldo Cruz Foundation, Rio de Janeiro, Brazil, between 2009 and 2017. The samples were obtained by biopsy of the active lesions using a 3–4 mm punch and fixed in 10% neutral buffered formalin and embedded in paraffin. The mycological culture technique for isolation of *Sporothrix* sp. was performed as described by Rippon (7). The DNA of type strain of *S. brasiliensis* CBS120339 (formerly IPEC 16490) (11) was used as positive control in every PCR assay. To monitor possible contamination, reaction mixtures without DNA were performed in the first and nested PCRs as negative controls.

### Preparation of FFPE Samples

Each paraffin block, including the blocks of negative control samples, was sectioned into two distinct paraffin sectioning protocols: (a) eight 5 μm thick sections (5 μm section) and (b) one 16 μm thick section (16 μm section), using a microtome. Weighing of the FFPE pellet samples was also performed in an analytical balance (Shimadzu, São Paulo, Brazil). The sections were submitted to DNA extraction from *Sporothrix* sp. as well as the other species of infectious agents (specificity and cross-reaction controls).

### DNA Extraction From FFPE Samples

DNA was extracted from paraffin sections using two different commercial DNA extraction kits: (1) QIAamp® DSP DNA FFPE Tissue Kit (Qiagen, California, USA) in which the sections of FFPE samples were deparaffinized with xylene (chemical DNA extraction); and (2) ReliaPrep^TM^ FFPE gDNA Miniprep System kit (Promega Corporation, Madison, USA) in which the sections of FFPE samples were deparaffinized with mineral oil and incubated at 80°C (thermal DNA extraction). The extraction steps followed all the manufacturer's instructions. After extraction, the extracted DNA was frozen at −30°C.

### DNA Quantification

The DNA quantification from all FFPE samples was evaluated in the DNA extract using two parameters: (1) DNA concentration, using spectrophotometric measurement of absorbance at 260 nm wavelength (NanoDrop™ 2000c, Thermo Fisher Scientific, Waltham, USA); and (2) DNA purity, using an absorbance ratio of 260 nm to 280 nm (A_260_/A_280_), considering the range of 1.8–2.0 to be of good quality (55).

### Nested PCR Assay

For the genus-level PCR identification of *Sporothrix* sp. in FFPE specimens, a nested PCR was performed according to a previously described method based on the amplification of the 18S ribosomal RNA (ITS) (40) with slight modifications (41).

A ready-to-use PCR master mix 2X solution was used (Promega Corporation, Madison, USA – Lot: 460191). PCR master mix contains 50 units/mL of Taq DNA polymerase supplied in a proprietary reaction buffer (pH 8.5), 400μM dATP, 400μM dGTP, 400μM dCTP, 400μM dTTP and 3mM MgCl2. For a 50 μL total reaction volume, reactions were standardized to obtain a final concentration of 100 to 120 ng of DNA. The reaction mixture of the first-round PCR consisted of 1–15 μL of DNA template, with final concentrations of 1X PCR master mix and 0, 2 μM of outer primers SS1 (5'-CTC GTT CGG CAC CTT ACA CG-3') and SS2 (5'-CGC TGC CAA AGC AAC GCG GG-3') (40). The reaction mixture of the nested PCR was identical, except that 3 μL of the first reaction product and the inner primer pair SS3 (5'-ACT CAC CAG GTC CAG ACA CGA TG-3') and SS4 (5'-CGC GGG CTA TTT AGC AGG TTA AG-3') (40) were used. The PCR reaction was performed in 0.5-mL thin-wall polypropylene tubes in a thermal cycler Veriti^TM^ 96-Well Thermal Cycler (Thermo Fisher Scientific, Massachusetts, USA) under the following conditions: initial denaturation at 95°C for 5 min, followed by 40 cycles of 1 min denaturation at 95°C, hybridization for 1 min at 68°C and extension for 1 min at 72°C, and then final extension for 10 min at 72°C. PCR products were analyzed by electrophoresis on a 2% w/v agarose gel (Agarose Ultra Pure^TM^, Thermo Fisher Scientific, Massachusetts, USA – Lot: 300647). DNA was stained with Blue Green Loading Dye I (LGC Biotecnologia, São Paulo, Brazil – Lot: 160919BT) and the amplicons were visualized on a LED K33-333 transilluminator (KASVI, Paraná, Brazil). The first round amplified a 305 bp fragment and the second one, a 152 bp fragment.

### Statistical Analysis

The exploratory analysis used median, interquartile range, minimum and maximum to summarize the quantitative variables and absolute and relative frequencies to describe the qualitative variables. To compare the quantitative variables, Wilcoxon Signed-Rank Test and Wilcoxon Rank Sum Test were used. The quantitative DNA purity variable was categorized in pure and not pure, considering pure values between 1.8 and 2.0 of the A_260_/A_280_ ratio, and is optimal around 1.8 (55). The sensitivity value of positive samples by nested PCR was provided with the respective 95% confidence interval (CI). *P* < 0.05 indicate significant differences in the tests, however, due to the sample size, whenever possible, uncertainty measures (variability and CI) were also provided. Free software R version 4.0 was used for statistical analyses.

## Results

The FFPE pellet samples were sectioned using both paraffin sectioning protocols (eight 5 μm thick sections and one 16 μm thick section). No statistical difference between the weights of the paraffin sectioning protocols was observed (*p* > 0.05). The median weight of the FFPE samples in the eight 5 μm thick sectioning protocol and in the single 16 μm thick section was 15.1 mg and 8.5 mg, respectively.

The comparison of the DNA extraction protocols in the FFPE mycological culture pellet samples based on the concentration and purity of the DNA using both paraffin sectioning protocols (5 and 16 μm) is shown in the Table 1A. There was no statistical difference in the DNA concentration between the extraction kits (*p* = 0.8203 in 5 μm and *p* = 1.000 in 16 μm). There was also no statistical difference in the DNA concentration between the tested sectioning protocols (Table 1A). The degree of purity of the extracted DNA was higher in the thermal DNA extraction protocol compared to the chemical extraction protocol using the 16 μm thick section (*p* = 0.009) (Table 1B).

**TABLE 1A:** Comparison of chemical and thermal DNA extraction protocols in six FFPE pellet samples of *Sporothrix* sp. and negative controls, using the two paraffin sectioning protocols (5 μm and 16 μm), in relation to the DNA concentration (ng/μL) and the degree of DNA purity (A_260_/A_280_ nm ratio).

**FFPE DNA extraction protocol**	**Parameter**	**5** **μm - Paraffin section**				**16** **μm - Paraffin section**				*p*-value^*^
		**Median**	**Min**.	**Max**.	**IQR**	**Median**	**Min**.	**Max**.	**IQR**	
**Chemical extraction**	DNA quantity (ng/μl)	8.2	4.5	122.8	13.5	8.9	0.6	152.8	13.7	0.4961
	DNA purity (A_260_/A_280_)	1.90	1.59	7.23	0.4	1.93	0.91	6.66	0.51	1.000
**Thermal extraction**	DNA quantity (ng/μl)	10.3	7.2	89.5	6.0	11.6	5.7	72.5	14.3	0.5703
	DNA purity (A_260_/A_280_)	1.71	1.49	1.92	0.09	1.63	1.33	1.99	0.42	1.000

*Median, minimum (min), maximum (max) values, and interquartile ranges (IQR) of the DNA quantification results are expressed*.

* *Wilcoxon test between the two paraffin sectioning protocols (5 μm and 16 μm)*.

**TABLE 1B:** Comparison of chemical and thermal DNA extraction protocols in six FFPE pellet samples of *Sporothrix* sp. and negative controls, using the two paraffin sectioning protocols, in relation to the degree of DNA purity (A_260_/A_280_ nm ratio).

**FFPE DNA extraction protocol**	**DNA purity**			
	**5** **μm**		**16** **μm**	
	**Pure (%)**	**Non-pure (%)**	**Pure (%)**	**Non-pure (%)**
**Chemical extraction**	2 (22.2)	7 (77.8)	2 (22.2)	7 (77.8)
**Thermal extraction**	2 (22.2)	7 (77.8)	4 (44.4)	5 (55.6)

*DNA results with the A_260_/A_280_ ratio falling within the range of 1.8–2.0 were considered as pure. The number of pure and/or non-pure samples and their percentage are expressed*.

**Figure 1:**
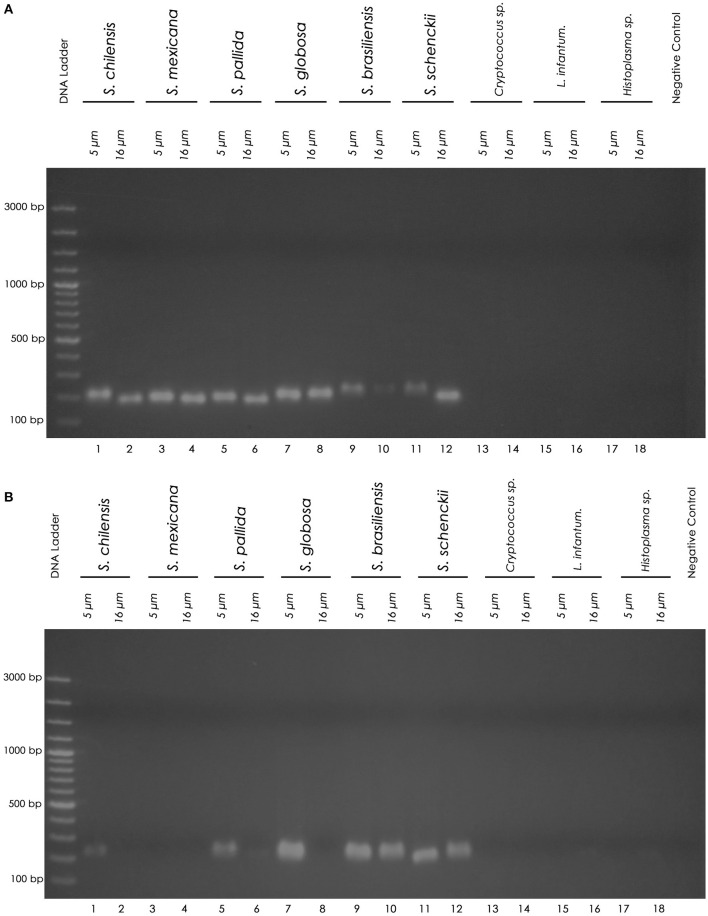
Nested PCR products on agarose gel. **(A)** Chemical DNA extraction. **(B)** Thermal DNA extraction. Left to right: Molecular marker DNA ladder, 100 bp (Fermentas). 1 (5 μm) and 2 (16 μm): *S. chilensis*; 3 (5 μm) and 4 (16 μm): *S. mexicana*; 5 (5 μm) and 6 (16 μm): *S. pallida*; 7 (5 μm) and 8 (16 μm): *S. globosa*; 9 (5 μm) and 10 (16 μm): *S. brasiliensis*; 11 (5 μm) and 12 (16 μm): *S. schenckii*; 13 (5 μm) and 14 (16 μm): *Cryptococcus* sp.; 15 (5 μm) and 16 (16 μm): *L. infantum*; 17 (5 μm) and 18 (16 μm): *Histoplasma* sp.; PCR negative control (sterile water). The nested PCR product is a 152 base pair (152 bp) amplicon.

Using the chemical DNA extraction protocol, nested PCR technique was able to detect the 152bp fragment of all six *Sporothrix* species tested in FFPE pellet samples using both paraffin sectioning protocols (100% sensitivity) (Figure 1). The sensitivity of the thermal extraction in the 5 and 16 μm sectioning protocols was 83 and 50%, respectively (Table 1C). Both FFPE DNA extraction protocols showed 100% specificity for *Sporothrix* sp. and no cross-reactions were observed, demonstrated by the absence of amplified product in nested PCR in FFPE samples of *Cryptococcus* sp., *Leishmania infantum* and *Histoplasma* sp. (Figure 1). In clinical FFPE samples from cats, the comparison of the DNA extraction protocols and the sensitivity of nested PCR are shown in Tables 2A–C. There was no statistical difference in the DNA concentration between the extraction kits in clinical samples (*p* = 0.0483 in 5 μm and *p* = 0.4316 in 16 μm). The nested PCR technique showed 50% sensitivity in chemical extraction and 30% sensitivity in thermal DNA extraction at the 5 μm thick sectioning protocol (Table 2C). Two clinical FFPE samples from 2009 and three from 2015 tested negative by nested PCR in all paraffin sectioning and DNA extraction protocols. Four samples from 2017 and one sample from 2015 amplified in at least one of the paraffin sectioning or DNA extraction protocols.

**TABLE 1C:** Comparison of chemical and thermal DNA extraction protocols in six FFPE pellet samples of *Sporothrix* sp. and negative controls, using the two paraffin sectioning protocols (5 μm and 16 μm), in relation to positivity, method sensitivity and confidence interval (95% CI).

**FFPE DNA extraction protocol**		**Nested PCR -** ***Sporothrix*** **sp**.			
		**Positive**	**Negative**	**Sensitivity (%)**	**95%CI (%)**
Chemical extraction	5 μm	6	0	100	54–100
	16 μm	6	0	100	54–100
Thermal extraction	5 μm	5	1	83.3	35.8–99.6
	16 μm	3	3	50	11.8–88.2

**TABLE 2A:** Comparison of chemical and thermal DNA extraction protocols in clinical FFPE skin samples from ten cats, using the two paraffin sectioning protocols (5 and 16 μm), in relation to the DNA concentration (ng/μL) and the degree of DNA purity (A_260_/A_280_ nm ratio).

**FFPE DNA extraction protocol**	**Parameter**	**5** **μm - Paraffin section**			**16** **μm - Paraffin section**			*p*-value*
		**Median**	**Min**.	**Max**.	**Median**	**Min**.	**Max**.	
**Chemical extraction**	DNA quantity (ng/μl)	11.05	0.50	65.0	6.70	0.10	68.60	0.4961
	DNA purity (A_260_/A_280_)	1.71	0	2.99	1.89	0	2.52	0.9375
**Thermal extraction**	DNA quantity (ng/μl)	18.25	2.14	73.70	13.30	2.90	49.20	0.1934
	DNA purity (A_260_/A_280_)	1.84	1.55	3.31	1.64	1.12	1.86	0.01953

Median, minimum (min), maximum (max) values, and interquartile ranges (IQR) of the DNA quantification results are expressed. *Wilcoxon test between the two paraffin sectioning protocols (5 μm and 16 μm).

**TABLE 2B:** Comparison of chemical and thermal DNA extraction protocols in clinical FFPE skin samples from ten cats, using the two paraffin sectioning protocols (5 and 16 μm), in relation to the degree of DNA purity (A_260_/A_280_ nm ratio).

**FFPE DNA extraction protocol**	**DNA purity**			
	**5** **μm**		**16** **μm**	
	**Pure (%)**	**Non-pure (%)**	**Pure (%)**	**Non-pure (%)**
**Chemical extraction**	1 (10)	9 (90)	5 (50)	5 (50)
**Thermal extraction**	5 (50)	5 (50)	2 (20)	8 (80)

*DNA results with the A_260_/A_280_ ratio falling within the range of 1.8 – 2.0 were considered as pure. The number of pure and/or non-pure samples and their percentage are expressed*.

**TABLE 2C:** Comparison of chemical and thermal DNA extraction protocols in clinical FFPE skin samples from ten cats, using the two paraffin sectioning protocols (5 and 16 μm), in relation to the positivity, method sensitivity and confidence interval (95% CI).

**FFPE DNA extraction protocol**		**Nested PCR -** ***Sporothrix*** **sp**.			
		**Positive**	**Negative**	**Sensitivity (%)**	**CI (%)**
**Chemical extraction**	5 μm	5	5	50	18.7–81.3
	16 μm	0	10	0	0–30.9
**Thermal extraction**	5 μm	3	7	30	6.7–65.2
	16 μm	0	10	0	0–30.9

## Discussion

FFPE tissues have several advantages for diagnostic purposes, as they are easy to handle and transport, their processing is low-cost and they can be stored for a long time at room temperature (26). However, obtaining DNA from this type of sample in sufficient quantity and quality using extraction techniques is a challenge (56). Several preanalytical factors can influence the analysis of nucleic acids in FFPE samples, such as biospecimen fixation, specimen size, block storage conditions, section thickness, section storage, and others (57). The 5 μm thick sections are commonly used in the analysis of different types of FFPE tissues (47, 58–63). The 16 μm thick sections were tested in this work as an alternative to produce samples with a smaller amount of paraffin, which contains PCR inhibitors (64), in an attempt to increase the sensitivity of the methods. In the present study, the chemical DNA extraction protocol demonstrated the best performance compared to the thermal protocol, mainly in 5 μm thick sectioning protocol.

Although the DNA concentration was similar in both extraction and paraffin sectioning protocols and the purest DNA was observed in thermal DNA extraction (16 μm section), the nested PCR assay demonstrated the superiority of the chemical DNA extraction protocol by the positivity observed in all FFPE pellet samples tested. The chemical extraction kit used in our study was also used by Sarnecka et al. (63) with FFPE tumor tissues. These authors obtained an amount of DNA similar to the present study (median of 13.20 ng/μL) and a DNA purity with the same median (A_260_/A_280_ = 1.90) in the 5 μm thick sectioning protocol. However, in the study of Sarnecka et al. (63), molecular analyzes were not performed and the performance of this kit was inferior to the other tested (automated extraction) (63). Higher DNA concentrations ranging from 12.3 to 618.9 ng were observed in another study with FFPE fungal samples using the same chemical DNA extraction kit with one 25 μm thick paraffin section and modifications such as longer incubation time with proteinase K and another cell lysis protocol (27).

In this work, the nested PCR methodology performed was similar to that described by Hu et al. (40) based on amplification of the 18S region of ribosomal RNA (ITS), using specific primers for *Sporothrix* sp. A semi-nested PCR methodology was developed for the diagnosis of human sporotrichosis in tissues embedded in paraffin with high sensitivity and specificity, but few cases were analyzed and there was a lack of details that make its reproducibility difficult (49). Also, the authors used the same chemical DNA extraction kit but it was not reported how the paraffin blocks were sectioned (49). Hu et al. (40) described a nested PCR assay for the detection of *S. schenckii* for diagnosing cutaneous sporotrichosis in frozen skin tissues (40). Based on them, Hayashi et al. (49) designed new primers that increased the sensitivity and specificity (100% and 98.7%, respectively) of their semi-nested PCR assay. In the present study, we used the same *Sporothrix* sp. specific primer sets, described by Hu et al. (40), and we were able to detect this fungus in FFPE samples.

Another important outcome in this work was the standardization of the methodology using a master mix solution to perform the PCR mixture, which facilitated the performance of the assays and the diagnosis of sporotrichosis in the clinical laboratory. The DNA yield from FFPE mycological culture pellet samples was considered ideal, as a standardization of the DNA concentration was performed in our study, but the highest degree of DNA purity was only 44.4%, within the purity criteria, observed in the thermal extraction protocol using the 16 μm sectioning protocol. This result may have been influenced by the low amount of material in the paraffin blocks. However, the nested PCR assay showed that even with low DNA purity in FFPE samples, it was possible to obtain high sensitivity and specificity. This indicates that in FFPE samples, the degree of DNA purity can be more variable than in fresh or frozen samples, with a range of purity larger than the one considered (A_260_/A_280_ = 1.8 – 2.0). Furthermore, the nested PCR was capable to identify at the genus-level all the pathogenic species of *Sporothrix* tested (*S. chilensis, S. mexicana, S. pallida, S. globosa, S. brasiliensis*and *S. schenckii*). Oliveira et al. (41) also used the same sets of primers to detect the *Sporothrix sensu lato* in cerebrospinal fluid, therefore, the nested amplification of the 18S rRNA gene fragment can detect all the *Sporothrix* species of the *Sporothrix* complex. In order to ensure no cross-reaction with other pathogens frequently found in feline and canine skin cases, *Cryptococcus* sp., *Leishmania infantum* and *Histoplasma* sp. FFPE tissue samples was included. As *Sporothrix* species have a large geographic distribution (65), this protocol can be applied in different regions of the world regardless of the species associated with the cases.

As Rio de Janeiro, Brazil is a hyperendemic region of sporotrichosis associated with feline zoonotic transmission, clinical FFPE samples from cats with sporotrichosis were used to validate our findings with the FFPE pellet samples. Some studies describe a high concentration of DNA extracted from paraffin blocks, but they differ in relation to the type of clinical sample tested, size of tissue embedded in paraffin, DNA extraction protocol and histological sections (66–69). Nechifor-Boilă et al. (67) considered the concentration of 100 ng/μL as an ideal cut-off point for a good DNA concentration in FFPE carcinoma samples. Our study with FFPE skin biopsy samples from cats with sporotrichosis showed a large variability in DNA concentration (0.10–73.70 ng/μL). However, our data corroborate with the previous study by Nechifor-Boilă et al. (67), since we validated our PCR method using a DNA concentration of 100–120 ng/μL, as the ideal one for the amplification of our target. In our validation study with clinical samples, the highest degree of purity was observed in FFPE tissues from cats using the chemical DNA extraction kit at the 16 μm paraffin sectioning protocol (50%) and the thermal DNA extraction kit at the 5 μm section (50%). Considering the clinical FFPE samples, negative results in the nested PCR were only observed in the oldest samples (from 2009 to 2015). Therefore, these negative results may have been influenced by the time of storage of these FFPE samples, because DNA degradation in FFPE samples increases considerably after 4–6 years of storage (70).

Despite the low DNA purity observed in the clinical samples, the nested PCR assay showed higher sensitivity in clinical FFPE samples from cats when using 5 μm paraffin sectioning protocol and the chemical extraction protocol (50% sensitivity). Ricci et al. (71) demonstrated PCR positivity of 74.2% in FFPE skin samples from patients with paracoccidioidomycosis, showing that in FFPE samples from skin lesions the sensitivity of the PCR technique is reduced. Our results were similar to Lysen et al. (70), who demonstrated PCR sensitivity of 54% for fungal identification from FFPE human tissues. Bernhardt et al. (47) detected DNA from *Sporothrix* in 2 of 52 (3.8%) FFPE skin samples from cats with histologically confirmed cutaneous and subcutaneous mycoses. Lau et al. (72) demonstrated higher sensitivity of a panfungal PCR assay in fresh frozen tissues compared to FFPE tissues for identifying different fungi in human and animal samples (fresh tissue: 96.8%; FFPE: 87.5% of culture-proven cases / fresh tissue: 100%; FFPE: 54.5% of histologically proven cases). The results of these authors (72) can be explained by the decreased quality, quantity, and size of DNA extracted from FFPE tissues compared to frozen tissues (73). However, FFPE samples can be preserved without refrigeration, making them easier to transport and store in areas with limited infrastructure and enable retrospective analysis (73, 74). In addition, due to maintenance, space, and labor requirements, storing FFPE tissues at room temperature for lengthy periods of time is more cost-effective than preserving frozen tissues at ultra-low temperatures (73, 74).

Despite the satisfactory results observed in our study by the nested PCR assay, it is necessary to search for alternatives to obtain FFPE samples with higher concentration and purity of DNA, such as the collection of a larger fragment from skin lesions of cats, in order to obtain a greater fungal load in these samples. Adjustments in DNA extraction protocols to reduce inhibitors and contaminants could also be pursued. Although we used specific primers for the amplification of the *Sporothrix* genus in combination with mycological culture, the standard reference method for the diagnosis of sporotrichosis, sequencing the amplicons would have improved our molecular analysis. Furthermore, the statistical tests may have been impaired due to the limited sample size.

In conclusion, the effective chemical DNA extraction of *Sporothrix* sp. DNA from FFPE samples using 5 μm thick paraffin sectioning protocol and the good sensitivity of the nested PCR assay indicates that the protocol herein presented have a great potential to be applied in *Sporothrix* sp. diagnosis in FFPE samples.

## Data Availability Statement

The original contributions presented in the study are included in the article/supplementary material, further inquiries can be directed to the corresponding authors.

## Ethics Statement

The animal study was reviewed and the protocols in this study were approved by the Animal Use Ethics Committee of the Oswaldo Cruz Foundation (CEUA-FIOCRUZ permit numbers L-041/06; LW-25/11; LW-32/12; LW-14/16; LW-12/17 and LW-17/17). This commission was established by FIOCRUZ Deliberative Council Resolution No. 004/2010 of January 21, 2010, and CIAEP No.463/2015 in compliance with National Council of Animal Experimentation Control (CONCEA).

## Author Contributions

RL, RM, and MO designed the study. RL carried out the experiments. RL, SP, and RM organized the database. RM and MO designed the methodology. RO performed the statistical analysis. SP, RO, and RM supervised the study. RM and MO raised funds. RL wrote the original draft. RL, RM, SP, RO, and MO reviewed and edited the manuscript. All authors contributed to the article and approved the submitted version.

## Funding

This study was supported by the State Funding Agency Fundação Carlos Chagas Filho de Amparo à Pesquisa do Estado do Rio de Janeiro (FAPERJ - Grants: JCNE E-26/201.433/2021; JCNE E-26/203.301/2017; E-26/201.737/2019), Conselho Nacional de Desenvolvimento Científico e Tecnológico (CNPq - Grant Proc. 409227/2016-1; Proc. 309682/2018-5; Proc. 312238/2020-7) and in part by the Coordenação de Aperfeiçoamento de Pessoal de Nível Superior - Brasil (CAPES) - Finance Code 001.

## Conflict of Interest

The authors declare that the research was conducted in the absence of any commercial or financial relationships that could be construed as a potential conflict of interest.

## Publisher's Note

All claims expressed in this article are solely those of the authors and do not necessarily represent those of their affiliated organizations, or those of the publisher, the editors and the reviewers. Any product that may be evaluated in this article, or claim that may be made by its manufacturer, is not guaranteed or endorsed by the publisher.

## References

[1] Díaz IAC. Epidemiology of sporotrichosis in Latin America. Mycopathologia. (1989). 108:113–6. 10.1007/BF00436061 2687693

[2] Arenas R. Micología Medica Ilustrada. 5th ed. Mexico city: McGraw-Hill (2014). p. 160–72.

[3] Gremião IDF Miranda LHM Reis EG Rodrigues AM Pereira SA. Zoonotic epidemic of sporotrichosis: cat to human transmission. PLoS Pathog. (2017) 13:e1006077. 10.1371/journal.ppat.1006077 28103311 PMC5245785

[4] Orofino-Costa R Rodrigues AM de Macedo PM Bernardes-Engemann AR. Sporotrichosis: an update on epidemiology, etiopathogenesis, laboratory and clinical therapeutics. An Bras Dermatol. (2017) 92:606–20. 10.1590/abd1806-4841.2017279 29166494 PMC5674690

[5] Gremião IDF Oliveira MME Monteiro de. Miranda LH, Saraiva Freitas DF, Pereira SA. Geographic Expansion of Sporotrichosis, Brazil. Emerg Infect Dis. (2020) 26:621–4. 10.3201/eid2603.190803 32091376 PMC7045854

[6] Pereira SA Gremião IDF Kitada AAB Boechat JS Viana PG Schubach TMP. The epidemiological scenario of feline sporotrichosis in Rio de Janeiro, State of Rio de Janeiro, Brazil. Rev Soc Bras Med Trop. (2014) 47:392–3. 10.1590/0037-8682-0092-2013 25075494

[7] Rippon JW. Medical Mycology: The Pathogenic Fungi and the Pathogenic Actinomycetes, 3rd ed. Philadelphia, PA: W B Saunders Co. (1988). p. 325–52.

[8] Barros MB de L Paes R de A Schubach AO. Sporothrix schenckii and Sporotrichosis. Clin Microbiol Rev. (2011) 24:633–54. 10.1128/CMR.00007-11 21976602 PMC3194828

[9] Dias NM Oliveira MME Portela MA Santos C Zancope-Oliveira RM Lima N. Sporotrichosis caused by *Sporothrix mexicana*, Portugal. Emerg Infect Dis. (2011) 17:1975–6. 10.3201/eid1710.110737 22000393 PMC3310684

[10] Madrid H Cano J Gené J Bonifaz A Toriello C Guarro J. Sporothrix globosa, a pathogenic fungus with widespread geographical distribution. Rev Iberoam Micol. (2009) 26:218–22. 10.1016/j.riam.2009.02.005 19635441

[11] Marimon R Cano J Gené J Sutton DA Kawasaki M Guarro J. Sporothrix brasiliensis, S. globosa, and S. mexicana, three new Sporothrix species of clinical interest. J Clin Microbiol. (2007) 45:3198–206. 10.1128/JCM.00808-07 17687013 PMC2045377

[12] Marimon R Genè J Cano J Guarro J. Sporothrix luriei: a rare fungus from clinical origin. Med Myco. (2008) 46:621–5. 10.1080/13693780801992837 19180753

[13] Oliveira MME de de Almeida-Paes R de Muniz MM de Barros MBL Galhardo MCG Zancope-Oliveira RM. Sporotrichosis caused by *sporothrix* globosa in Rio De Janeiro, Brazil: Case report. Mycopathologia. (2010) 169:359–63. 10.1007/s11046-010-9276-7 20131099

[14] Rodrigues AM de Melo Teixeira M de Hoog GS Schubach TMP Pereira SA Fernandes GF. Phylogenetic Analysis Reveals a High Prevalence of *Sporothrix brasiliensis* in Feline sporotrichosis outbreaks. PLoS Negl Trop Dis. (2013) 7:e2281. 10.1371/journal.pntd.0002281 23818999 PMC3688539

[15] Rodrigues AM Cruz Choappa R Fernandes GF de Hoog GS de Camargo ZP. Sporothrix chilensis sp. nov. (Ascomycota: Ophiostomatales), a soil-borne agent of human sporotrichosis with mild-pathogenic potential to mammals. Fungal Biol. (2016) 120:246–64. 10.1016/j.funbio.2015.05.006 26781380

[16] Kano R Okubo M Siew HH Kamata H Hasegawa A. Molecular typing of *Sporothrix schenckii* isolates from cats in Malaysia. Mycoses. (2015) 58:220–4. 10.1111/myc.12302 25727965

[17] Montenegro H Rodrigues AM Dias MAG da Silva EA Bernardi F de Camargo ZP. Feline sporotrichosis due to *Sporothrix brasiliensis*: an emerging animal infection in São Paulo, Brazil. BMC Vet Res. (2014) 10:269. 10.1186/s12917-014-0269-5 25407096 PMC4244058

[18] Song Y Li SS Zhong SX Liu YY Yao L Huo SS. Report of 457 sporotrichosis cases from Jilin province, northeast China, a serious endemic region. J Eur Acad Dermatology Venereol. (2013) 27:313–8. 10.1111/j.1468-3083.2011.04389.x 22176524

[19] Macêdo-Sales PA Souto SRLS Destefani CA Lucena RP Luiz R Machado D. Domestic feline contribution in the transmission of Sporothrix in Rio de Janeiro State, Brazil: a comparison between infected and non-infected populations. BMC Vet Res. (2018) 14:19. 10.1186/s12917-018-1340-4 29347940 PMC5774141

[20] Boechat JS Oliveira MME Almeida-Paes R Gremião IDF Machado AC de S Oliveira R de VC. Feline sporotrichosis: associations between clinical-epidemiological profiles and phenotypic-genotypic characteristics of the etiological agents in the Rio de Janeiro epizootic area. Mem Inst Oswaldo Cruz. (2018) 113:185–96. 10.1590/0074-02760170407 29412358 PMC5804311

[21] Etchecopaz A Toscanini MA Gisbert A Mas J Scarpa M Iovannitti CA. Sporothrix brasiliensis: a review of an emerging south american fungal pathogen, its related disease, presentation and spread in Argentina. J Fungi. (2021) 7:1–33. 10.3390/jof7030170 33652625 PMC7996880

[22] Rossow JA Queiroz-Telles F Caceres DH Beer KD Jackson BR Pereira JG. A one health approach to combatting *Sporothrix brasiliensis*: narrative review of an emerging zoonotic fungal pathogen in south america. J Fungi. (2020) 6:1–27. 10.3390/jof6040247 33114609 PMC7712324

[23] Zancope-Oliveira RM Almeida-Paes R de Oliveira MME Freitas DFS Galhardo MCG. New diagnostic applications in sporotrichosis. In: Khopkar U, editor, Skin Biopsy - Perspectives. InTech (2011). Available online at: http://www.intechopen.com/journals/skin-biopsy-perspectives/new-diagnostic-applications-insporotrichosis

[24] Alexander BD Pfaller MA. Contemporary tools for the diagnosis and management of invasive mycoses. Clin Infect Dis. (2006) 43(Suppl 1):S15–27. 10.1086/504491

[25] Rodrigues AM de Hoog GS de Camargo ZP. Molecular diagnosis of pathogenic *Sporothrix* species. PLoS Negl Trop Dis. (2015) 9:1–22. 10.1371/journal.pntd.0004190 26623643 PMC4666615

[26] Donczo B Guttman A. Biomedical analysis of formalin-fixed, paraffin-embedded tissue samples: the holy grail for molecular diagnostics. J Pharm Biomed Anal. (2018) 155:125–34. 10.1016/j.jpba.2018.03.065 29627729

[27] Frickmann H Loderstaedt U Racz P Tenner-Racz K Eggert P Haeupler A. Detection of tropical fungi in formalin-fixed, paraffin-embedded tissue: Still an indication for microscopy in times of sequence-based diagnosis? Biomed Res Int. (2015) 2015:938721. 10.1155/2015/938721 25961048 PMC4417575

[28] Lopes-bezerra LM Mora-montes HM Zhang Y Nino-vega G Rodrigues AM Camargo ZP De. Sporotrichosis between 1898 and 2017 : the evolution of knowledge on a changeable disease and on emerging etiological agents. (2018) 56:126–43. 10.1093/mmy/myx103 29538731

[29] Silva JN Miranda LHM Menezes RC Gremião IDF Oliveira RVC Vieira SMM. Comparison of the sensitivity of three methods for the early diagnosis of sporotrichosis in cats. J Comp Pathol. (2018) 160:72–8. 10.1016/j.jcpa.2018.03.002 29729723

[30] Arenas R Miller D Campos-Macias P. Epidemiological data and molecular characterization (mtDNA) of *Sporothrix schenckii* in 13 cases from Mexico. Int J Dermatol. (2007) 46:177–9. 10.1111/j.1365-4632.2006.03036.x 17269971

[31] Rodrigues AM De Hoog GS De Camargo ZP. Genotyping species of the *Sporothrix schenckii* complex by PCR-RFLP of calmodulin. Diagn Microbiol Infect Dis. (2014) 78:383–7. 10.1016/j.diagmicrobio.2014.01.004 24525143

[32] Mesa-Arango AC Reyes-Montes M del R Pérez-Mejía A Navarro-Barranco H Souza V Zúñiga G. Phenotyping and genotyping of *Sporothrix schenckii* isolates according to geographic origin and clinical form of sporotrichosis. J Clin Microbiol. (2002) 40:3004–11. 10.1128/JCM.40.8.3004-3011.2002 12149366 PMC120692

[33] Oliveira MME de Sampaio P Almeida-Paes R Pais C Gutierrez-Galhardo MC Zancope-Oliveira RM. Rapid identification of *Sporothrix* species by T3B fingerprinting. J Clin Microbiol. (2012) 50:2159–62. 10.1128/JCM.00450-12 22403427 PMC3372112

[34] Oliveira MME Almeida-Paes R Gutierrez-Galhardo MC Zancope-Oliveira RM. Molecular identification of the *Sporothrix schenckii* complex. Rev Iberoam Micol. (2014) 31:2–6. 10.1016/j.riam.2013.09.008 24270070

[35] Rodrigues AM De Hoog G Zhang Y De Camargo ZP. Emerging sporotrichosis is driven by clonal and recombinant *Sporothrix* species. Emerg Microbes Infect. (2014) 3:e32. 10.1038/emi.2014.33 26038739 PMC4051365

[36] Rashmi MV Hamsaveena. Global ITS diversity in the *Sporothrix schenckii* complex. Malays J Pathol. (2015) 37:247–51. 10.1007/s13225-013-0220-2 26712670

[37] Marimon R Gené J Cano J Trilles L Lazéra MDS Guarro J. Molecular phylogeny of *Sporothrix schenckii*. J Clin Microbiol. (2006) 44:3251–6. 10.1128/JCM.00081-06 16954256 PMC1594699

[38] Zhang Y Hagen F Stielow B Rodrigues AM Samerpitak K Zhou X. Phylogeography and evolutionary patterns in *Sporothrix* spanning more than 14 000 human and animal case reports. Persoonia. (2015) 35:1. 10.3767/003158515X687416 26823625 PMC4713101

[39] Kano R Nakamura Y Watanabe S Tsujimoto H Hasegawa A. Chitinsynthase– O. Identification of Sporothrix schenckii based on sequences of the chitin synthase 1 gene. Mycoses. (2001) 265:261–5. 10.1111/j.1439-0507.2001.00655.x 11714059

[40] Hu S Chung WH Hung SI Ho HC Wang ZW Chen CH. Detection of *Sporothrix schenckii* in clinical samples by a nested PCR assay. J Clin Microbiol. (2003) 41:1414–8. 10.1128/JCM.41.4.1414-1418.2003 12682123 PMC153868

[41] Oliveira MME Muniz M de M Almeida-Paes R Zancope-Oliveira RM Freitas AD Lima MA. Cerebrospinal fluid PCR: A new approach for the diagnosis of CNS sporotrichosis. PLoS Negl Trop Dis. (2020) 14:1–4. 10.1371/journal.pntd.0008196 32673308 PMC7365394

[42] Kanbe T Natsume L Goto I Kawasaki M Mochizuki T Ishizaki H. Rapid and specific identification of *Sporothrix schenckii* by PCR targeting the DNA topoisomerase II gene. J Dermatol Sci. (2005) 38:99–106. 10.1016/j.jdermsci.2004.12.024 15862942

[43] Kourkoumpetis TK Fuchs BB Coleman JJ Desalermos A Mylonakis E. Polymerase chain reaction-based assays for the diagnosis of invasive fungal infections. Clin Infect Dis. (2012) 54:1322–31. 10.1093/cid/cis132 22362884 PMC3491854

[44] Macêdo-Sales PA Souza LOP Della-Terra PP Lozoya-Pérez NE Machado RLD Rocha EM da S da. Coinfection of domestic felines by distinct *Sporothrix brasiliensis* in the Brazilian sporotrichosis hyperendemic area. Fungal Genet Biol. (2020) 140:103397. 10.1016/j.fgb.2020.103397 32325170

[45] Gonsales FF Fernandes NCCA Mansho W Montenegro H Benites NR. Direct PCR of lesions suggestive of sporotrichosis in felines. Arq Bras Med Veterinária e Zootec. (2020) 72:2002–6. 10.1590/1678-4162-11743

[46] Kano R Watanabe K Murakami M Yanai T Hasegawa A. Molecular diagnosis of feline sporotrichosis. Vet Rec. (2005) 156:484–5. 10.1136/vr.156.15.484 15828746

[47] Bernhardt A von Bomhard W Antweiler E Tintelnot K. Molecular identification of fungal pathogens in nodular skin lesions of cats. Med Mycol. (2015) 53:132–44. 10.1093/mmy/myu082 25550386

[48] Cao W Hashibe M Rao JY Morgenstern H Zhang ZF. Comparison of methods for DNA extraction from paraffin-embedded tissues and buccal cells. Cancer Detect Prev. (2003) 27:397–404. 10.1016/S0361-090X(03)00103-X 14585327

[49] Hayashi S Kaminaga T Baba A Koike S Koike M Kanno M. Diagnostic value of a nested polymerase chain reaction for diagnosing cutaneous sporotrichosis from paraffin-embedded skin tissue. Mycoses. (2019) 62:1148–53. 10.1111/myc.13004 31518455

[50] Koike M Hayashi S Baba A Koike S Gonmori T Kaminaga T. PCR-based diagnosis of *Sporothrix* infection using DNA from paraffin-embedded skin specimens in previously undiagnosed cases. Eur J Dermatol. (2020) 30:614–5. 10.1684/ejd.2020.3862 32959783 PMC8120491

[51] Valeriano CAT de Lima-Neto RG Inácio CP Rabello VB de S Oliveira EP Zancopé-Oliveira RM. Is *Sporothrix chilensis* circulating outside Chile? PLoS Negl Trop Dis. (2020) 14:e0008151. 10.1371/journal.pntd.0008151 32226021 PMC7162539

[52] Oliveira MME Almeida-Paes R Muniz MM Gutierrez-Galhardo MC Zancope-Oliveira RM. Phenotypic and molecular identification of sporothrix isolates from an epidemic area of sporotrichosis in Brazil. Mycopathologia. (2011) 172:257–67. 10.1007/s11046-011-9437-3 21701792

[53] D'Alessandro E Giosa D Huang L Zhang J Gao W Brankovics B. Draft genome sequence of the dimorphic fungus *Sporothrix pallida*, a nonpathogenic species belonging to sporothrix, a genus containing agents of human and feline sporotrichosis. Genome Announc. (2016) 4:184–200. 10.1128/genomeA.00184-16 27034494 PMC4816622

[54] Carson F Cappellano CH. Histotechnology: A Self Instructional Text. 4th ed. American Society for Clinical Pathologyn Chicago (2015).

[55] Thermo Fisher Scientific. Nucleic Acid - Thermo Scientific NanoDrop Spectrophotometers. Nucleic Acid. (2010) 11:1–30.

[56] Maraschin BJ da Silva VP Rock L Sun H Visioli F Rados PV. Optimizing fixation protocols to improve molecular analysis from FFPE tissues. Braz Dent J. (2017) 28:82–4. 10.1590/0103-6440201701211 28301023

[57] Bass BP Engel KB Greytak SR Moore HM. A review of preanalytical factors affecting molecular, protein, and morphological analysis of Formalin-Fixed, Paraffin-Embedded (FFPE) tissue: how well do you know your FFPE specimen? Arch Pathol Lab Med. (2014) 138:1520–30. 10.5858/arpa.2013-0691-RA 25357115

[58] Barton Rogers B Alpert LC Hine EAS Buffone GJ. Analysis of DNA in fresh and fixed tissue by the polymerase chain reaction. Am J Pathol. (1990) 136:541–8. 2156429 PMC1877479

[59] Isola J DeVries S Chu L Ghazvini S Waldman F. Analysis of changes in DNA sequence copy number by comparative genomic hybridization in archival paraffin-embedded tumor samples. Am J Pathol. (1994) 145:1301–8. 7992835 PMC1887491

[60] Janecka A Adamczyk A Gasińska A. Comparison of eight commercially available kits for DNA extraction from formalin-fixed paraffin-embedded tissues. Anal Biochem. (2015) 476:8–10. 10.1016/j.ab.2015.01.019 25640584

[61] Lin J Kennedy SH Svarovsky T Rogers J Kemnitz JW Xu A. High-quality genomic DNA extraction from formalin-fixed and paraffin-embedded samples deparaffinized using mineral oil. Anal Biochem. (2009) 395:265–7. 10.1016/j.ab.2009.08.016 19698695 PMC2764035

[62] Muñoz-Cadavid C Rudd S Zaki SR Patel M Moser SA Brandt ME. Improving molecular detection of fungal DNA in formalin-fixed paraffin-embedded tissues: Comparison of five tissue DNA extraction methods using panfungal PCR. J Clin Microbiol. (2010) 48:2147–53. 10.1128/JCM.00459-10 20392915 PMC2884522

[63] Sarnecka AK Nawrat D Piwowar M Ligeza J Swadzba J Wójcik P. extraction from FFPE tissue samples – a comparison of three procedures. Wspolczesna Onkol. (2019) 23:52–8. 10.5114/wo.2019.83875 31061638 PMC6500389

[64] Frank TS Svoboda-Newman SM Hsi ED. Comparison of methods for extracting DNA from formalin-fixed paraffin sections for nonisotopic PCR. Diagnostic Mol Pathol. (1996) 5:220–4. 10.1097/00019606-199609000-00012 8866237

[65] Rodrigues AM Della Terra PP Gremião ID Pereira SA Orofino-Costa R de Camargo ZP. The threat of emerging and re-emerging pathogenic *Sporothrix* species. Mycopathologia. (2020) 185:813–42. 10.1007/s11046-020-00425-0 32052359

[66] Nagahashi M Shimada Y Ichikawa H Nakagawa S Sato N Kaneko K. Formalin-fixed paraffin-embedded sample conditions for deep next generation sequencing. J Surg Res. (2017) 220:125–32. 10.1016/j.jss.2017.06.077 29180174 PMC5726294

[67] Nechifor-Boilă AC Loghin A Vacariu V Halatiu VB Borda A. The storage period of the formalin-fixed paraffin-embedded tumor blocks does not influence the concentration and purity of the isolated DNA in a series of 83 renal and thyroid carcinomas. Rom J Morphol Embryol. (2015) 56:759–63. 26429169

[68] Patel PG Selvarajah S Boursalie S How NE Ejdelman J Guerard KP. Preparation of formalin-fixed paraffin-embedded tissue cores for both RNA and DNA extraction. J Vis Exp. (2016) 54299. 10.3791/54299 27583817 PMC5091935

[69] Miranda LHM Quintella LP dos Santos IB Menezes RC Figueiredo FB Gremião IDF. Histopathology of canine sporotrichosis: a morphological study of 86 cases from Rio de Janeiro (2001-2007). Mycopathologia. (2009) 168:79–87. 10.1007/s11046-009-9198-4 19360480

[70] Lysen C Silva-Flannery L Zaki SR Gary JM Lockhart SR. Performance evaluation of fungal DNA PCR amplification from formalin-fixed paraffin-embedded tissue for diagnosis: Experience of a tertiary reference laboratory. Mycoses. (2021) 64:603–11. 10.1111/myc.13249 33527526 PMC11950817

[71] Ricci G Campanini EB Nishikaku AS Ester M Marques A Ladr RB. PbGP43 genotyping using paraffin-embedded biopsies of human paracoccidioidomycosis reveals a genetically distinct lineage in the *Paracoccidioides Brasiliensis* Complex. Mycopathologia. (2021). 10.1007/s11046-021-00608-3. [Epub ahead of print]. 34870754

[72] Lau A Chen S Sorrell T Carter D Malik R Martin P. Development and clinical application of a panfungal PCR assay to detect and identify fungal DNA in tissue specimens. J Clin Microbiol. (2007) 45:380–5. 10.1128/JCM.01862-06 17122000 PMC1829013

[73] Maes RK Langohr IM Wise AG Smedley RC Thaiwong T Kiupel M. Beyond H&E: integration of nucleic acid-based analyses into diagnostic pathology. Vet Pathol. (2014) 51:238–56. 10.1177/0300985813505878 24129897

[74] Kokkat TJ Patel MS McGarvey D LiVolsi VA Baloch ZW. Archived Formalin-Fixed Paraffin-Embedded (FFPE) blocks: a valuable underexploited resource for extraction of DNA, RNA, and Protein. Biopreserv Biobank. (2013) 11:101–6. 10.1089/bio.2012.0052 24845430 PMC4077003

